# Pharmacokinetics of prolonged infusion of high-dose dexmedetomidine in critically ill patients

**DOI:** 10.1186/cc10518

**Published:** 2011-10-26

**Authors:** Timo Iirola, Riku Aantaa, Ruut Laitio, Erkki Kentala, Maria Lahtinen, Andrew Wighton, Chris Garratt, Tuula Ahtola-Sätilä, Klaus T Olkkola

**Affiliations:** 1 Department of Anaesthesiology, Intensive Care, Emergency Care and Pain Medicine, University of Turku and Turku University Hospital, PO Box 52, FI-20521 Turku, Finland; 2 Clinical Research Services Turku (CRST), University of Turku, CRST, FI-20014 University of Turku, Finland; 3 Orion Pharma R&D, PO Box 6792, Nottingham, NG1 1AH, UK; 4 Orion Pharma, PO Box 65, FI-02101 Espoo, Finland

## Abstract

**Introduction:**

Only limited information exists on the pharmacokinetics of prolonged (> 24 hours) and high-dose dexmedetomidine infusions in critically ill patients. The aim of this study was to characterize the pharmacokinetics of long dexmedetomidine infusions and to assess the dose linearity of high doses. Additionally, we wanted to quantify for the first time in humans the concentrations of H-3, a practically inactive metabolite of dexmedetomidine.

**Methods:**

Thirteen intensive care patients with mean age of 57 years and Simplified Acute Physiology Score (SAPS) II score of 45 were included in the study. Dexmedetomidine infusion was commenced by using a constant infusion rate for the first 12 hours. After the first 12 hours, the infusion rate of dexmedetomidine was titrated between 0.1 and 2.5 μg/kg/h by using predefined dose levels to maintain sedation in the range of 0 to -3 on the Richmond Agitation-Sedation Scale. Dexmedetomidine was continued as long as required to a maximum of 14 days. Plasma dexmedetomidine and H-3 metabolite concentrations were measured, and pharmacokinetic variables were calculated with standard noncompartmental methods. Safety and tolerability were assessed by adverse events, cardiovascular signs, and laboratory tests.

**Results:**

The following geometric mean values (coefficient of variation) were calculated: length of infusion, 92 hours (117%); dexmedetomidine clearance, 39.7 L/h (41%); elimination half-life, 3.7 hours (38%); and volume of distribution during the elimination phase, 223 L (35%). Altogether, 116 steady-state concentrations were found in 12 subjects. The geometric mean value for clearance at steady state was 53.1 L/h (55%). A statistically significant linear relation (*r*^2 ^= 0.95; *P *< 0.001) was found between the areas under the dexmedetomidine plasma concentration-time curves and cumulative doses of dexmedetomidine. The elimination half-life of H-3 was 9.1 hours (37%). The ratio of AUC_0-∞ _of H-3 metabolite to that of dexmedetomidine was 1.47 (105%), ranging from 0.29 to 4.4. The ratio was not statistically significantly related to the total dose of dexmedetomidine or the duration of the infusion.

**Conclusions:**

The results suggest linear pharmacokinetics of dexmedetomidine up to the dose of 2.5 μg/kg/h. Despite the high dose and prolonged infusions, safety findings were as expected for dexmedetomidine and the patient population.

**Trial Registration:**

ClinicalTrials.gov: NCT00747721

## Introduction

Many patients treated in intensive care units (ICUs) require sedation and analgesia to tolerate the tracheal tube, mechanical ventilation, and other intensive care procedures. However, commonly used sedatives like propofol, benzodiazepines, and opioids also have potential adverse effects that may increase morbidity and prolong the patients' clinical course [1]. Consequently, new drugs, with alternate mechanisms of action, have been developed for sedation of ICU patients.

**Figure 1:**
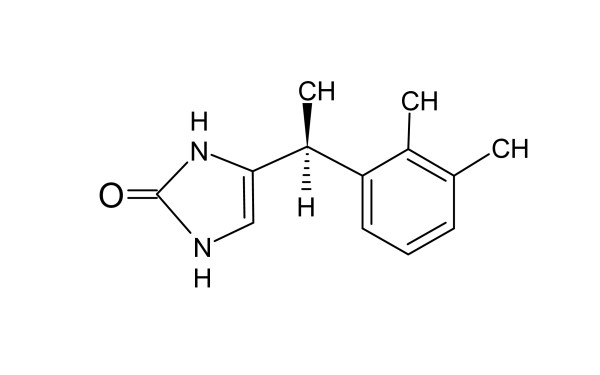
**Structural formula of H-3 metabolite**.

Dexmedetomidine is a highly selective and potent α-2 adrenoreceptor agonist indicated for procedural and ICU sedation. It is quickly distributed into tissues with a distribution half-life of approximately 6 minutes. It is metabolized in the liver to practically inactive products, mainly glucuronides, with a mean elimination half-life of 2 to 2.5 hours [2], with an extraction ratio of 0.71 [3]. Direct *N*-glucuronidation accounts for one third of the metabolism of dexmedetomidine [2]. Additionally, the drug is metabolized by multiple cytochrome P450 enzymes, especially CYP2A6, but also CYP1A2, CYP2C19, CYP2D6, and CYP2E1 [4]. Approximately 90% of an administered human drug dose is excreted as metabolites in the urine, and 10%, in the feces [2]. As well as offering sedation and anxiolysis, dexmedetomidine has moderate analgesic qualities, and it reduces the stress response to noxious stimuli [5]. Although the pharmacokinetics of dexmedetomidine have been studied previously during short-term use in an ICU setting [6], to our knowledge, only limited information exists on the pharmacokinetics of prolonged (> 24 hours) and high-dose dexmedetomidine infusions in critically ill patients. This study was designed to characterize the pharmacokinetics of long dexmedetomidine infusions and specifically to assess the dose linearity of high doses. We also quantified for the first time in humans the concentrations of the previously poorly characterized H-3 metabolite of dexmedetomidine (Figure 1). Because the structure of the H-3 metabolite has been unknown, no bioanalytic methods have been available until quite recently. The metabolite has practically no relevant pharmacologic activity (unpublished data).

## Materials and methods

### Patients

This study (EudraCT number 2008-001646-10/ClinicalTrials.gov identifier NCT00747721) was conducted in a 24-bed mixed intensive care unit of a tertiary teaching hospital in Finland from September 2008 to February 2009. It was approved by the Ethics Committee of the Hospital District of Southwest Finland and by the Finnish Medicines Agency. It was conducted according to the revised Declaration of Helsinki of the World Medical Association and ICH GCP guidelines for good clinical trial practice with external study monitoring.

On empiric grounds, it was planned to recruit between 12 and 20 patients so that at least six of the patients would have a minimum duration of 5 days of dexmedetomidine infusion. In total, 13 patients were enrolled. A written informed consent was obtained from the legal representatives of potential study subjects, and the decision for entry was made according to the inclusion and exclusion criteria. Patients older than 18 years, needing light to moderate sedation (0 to -3 on the Richmond Agitation-Sedation Scale (RASS) [7]) for at least 24 hours to tolerate the ventilator, were eligible for the study. Patients having an acute severe intracranial or spinal neurologic disorder, uncompensated circulatory failure (mean arterial pressure < 55 mm Hg despite vasopressors and inotropes), severe bradycardia, second- or third-degree atrioventricular block, severe hepatic impairment, positive pregnancy test, needing continuous muscle relaxation, using centrally acting α-2 agonists or antagonists, or having an allergy to dexmedetomidine or any excipients of the study treatment were not considered eligible for the study.

### Study treatment

No loading dose was administered, but a constant intravenous infusion of dexmedetomidine was given for an initial period of 12 hours. The starting dose was based on our previous experiences with sedative agents in the ICU and determined by the pre-study dose of propofol or midazolam: a dose of 0.1, 0.2, 0.45, or 0.7 μg/kg/h of dexmedetomidine was considered to be equivalent to < 0.3, 0.3 to 0.79, 0.8 to 1.59, and > 1.6 mg/kg/h of propofol or < 0.03, 0.03 to 0.06, 0.06 to 0.09, and > 0.09 mg/kg/h of midazolam, respectively. Downward titration was allowed in case of oversedation or an adverse event. After the 12-hour constant-rate infusion, the infusion rate of dexmedetomidine was titrated as needed between a minimum of 0.1 and a maximum of 2.5 μg/kg/h by titrating the dose stepwise upward or downward to maintain sedation in range of 0 to -3 by using the RASS. Only predefined dose levels of 0.1, 0.2, 0.45, 0.7, 0.95, 1.2, 1.4, 1.7, 2.1, and 2.5 μg/kg/h of dexmedetomidine were used. Propofol or midazolam was discontinued on commencement of dexmedetomidine infusion. However, the patient was allowed to be given additional sedation with the previous sedative agent (propofol or midazolam) if the target RASS of 0 to -3 was not achieved with dexmedetomidine.

Dexmedetomidine was continued as long as required to a maximum of 14 days. When the decision to discontinue the treatment was taken, it had to be stopped abruptly and not to be commenced again. If further sedation was required after dexmedetomidine had been stopped, standard-of-care sedation was instituted. After completion of the study treatment, study subjects were monitored for 48 hours and contacted by telephone or visit 31 and 45 days after starting the study treatment to record possible adverse events and concomitant treatments related to serious adverse events.

### Concomitant treatments

Oxycodone and fentanyl were used for the treatment of pain. The co-administration of centrally acting α-2 agonists or antagonists was prohibited. All concomitant treatments from screening to the end of the 48-hour follow-up period were recorded.

### Blood sampling

Arterial or central venous blood samples for dexmedetomidine and H-3 metabolite concentration measurements were collected before and 0.25, 0.5, 0.75, 1, 2, 3, 4, 6, 8, and 12 hours after starting study treatment, 3 times per day in the morning, afternoon, and evening of the following treatment days. Additional samples were drawn immediately before and 0.25, 0.5, 0.75, 1, 2, 3, 4, 6, 9, 12, 15, 18, 24, 36, and 48 hours after stopping study treatment. In case of hemodiafiltration, a venous blood sample was taken before commencing filtration. Thereafter, concomitant venous samples from the afferent and efferent limbs of the filter circuit were withdrawn at 2, 4, 6, and 8 hours.

### Sample handling

The 3-ml blood samples were drawn into chilled EDTA tubes, and they were immediately put into crushed ice. The samples were delivered in a few minutes to an accredited laboratory, where they were stored appropriately and centrifuged at 4°C within 3 hours after sampling. For logistic reasons, the plasma was frozen immediately after centrifugation at -20°C, and the samples were transferred to -70°C within 72 hours, where they were stored until the analysis.

### Dexmedetomidine and H-3 metabolite analysis

Concentrations of dexmedetomidine and H-3 metabolite in EDTA plasma samples were determined with high-performance liquid chromatography and mass spectrometric detection (HPLC-MS/MS; Shimadzu Prominence HPLC, Kyoto, Japan) connected to an AB Sciex API4000 mass spectrometer (Toronto, Ontario, Canada), as previously described [8]. The mobile phase was 0.1% formic acid in a mixture of 1:1:1 (vol/vol/vol) methanol/acetonitrile/water. The lower limit of quantification was 0.02 ng/ml. The within- and between-run precision of the assay (coefficient of variation) was within 7.5% in the relevant concentration range. The data from the HPLC-MS/MS analyses were collected by using Analyst 1.4.1 software (AB Sciex, Toronto, Ontario, Canada). The calculations were based on peak area ratios of the analyte and the internal standard. Deuterated medetomidine (Orion Pharma, Espoo, Finland) and H-3 metabolite (Orion Pharma) were used as the internal standards.

### Pharmacokinetic analysis

The pharmacokinetics of dexmedetomidine and the H-3 metabolite were characterized by using noncompartmental methods with the pharmacokinetic program WinNonlin 5.0.1 Professional (Pharsight Corporation, Mountain View, CA, USA). For each subject, the terminal log-linear phase of the plasma drug concentration-time curve for postinfusion data was identified visually, and the elimination rate constant (*k*_e_) was determined by regression analysis. The elimination half-life (*t*_1/2_) was calculated from the equation *t*_1/2 _= ln 2/*k*_e_. Plasma clearance (Cl) and volume of distribution during elimination (V) were calculated by the use of standard noncompartmental methods based on statistical moment theory. The areas under the drug plasma concentration-time curves (AUC_0-∞_) were estimated by using the trapezoidal method with extrapolation to infinity. In patients lacking the data during the 48-hour follow-up period after stopping dexmedetomidine infusion, the AUC_0-∞ _was estimated by using the mean *k*_e _value of the other subjects, as follows: AUC_0-∞ _= AUC_0-last _+ last measured concentration/mean *k*_e_. During the continuous infusion of dexmedetomidine, we estimated repetitively dexmedetomidine Cl_ss _from Cl_ss _= I_ss_/C_ss_, where I_ss _= infusion rate at steady state and C_ss _= concentration at steady state. It was estimated that steady state was reached after a constant infusion of 15 hours, which clearly exceeds 3.3 half-lives, generally assumed to be necessary for reaching steady state during dexmedetomidine infusion [2,9].

### Analysis of sedation

Depth of sedation was assessed at approximately at the same time each day during the treatment and the 48-hour follow-up period by using the RASS.

### Assessment of safety and tolerability

Adverse events, heart rate (HR), mean arterial blood pressure (MAP), 12-lead ECG, continuous ECG, and safety laboratory assessments were used as safety and tolerability variables. Adverse events and serious adverse events were defined as suggested by the European Medicines Agency [10]. In addition to clinically significant changes in vital signs, sustained MAP lower than 50 mm Hg or higher than 125 mm Hg and HR lower than 50 beats/min or higher than 120 beats/min (provided change in HR was ≥ 10%) were reported as adverse events.

Cardiac rhythm, including arrhythmias and AV blocks, was followed continuously and recorded daily. Any clinically significant new abnormalities since the last report were reported as adverse events. In addition, any changes indicative of myocardial ischemia were reported as adverse events.

Blood samples were collected for complete blood count, glucose, urea, creatinine, albumin, alanine aminotransferase, aspartate aminotransferase, γ-glutamyl transferase, alkaline phosphatase, bilirubin, lactate dehydrogenase, troponin T, total protein, triglycerides, total cholesterol, sodium, potassium, calcium, chloride, and cortisol. Changes in the laboratory parameters resulting to a new diagnosis or a change in the treatment were considered to be clinically significant and were reported as adverse events. In addition, elevated troponin T indicative of myocardial ischemia, liver enzymes > 5 times the upper limit of normal, and bilirubin > 5 times the upper limit of normal were reported as adverse events.

### Statistical methods

All analyses and tabulations were performed in treated subjects and are presented as geometric mean (coefficient of variation), unless otherwise indicated. The relations of the dexmedetomidine Cl and creatinine, and the total dexmedetomidine dose, and the dexmedetomidine AUC_0-∞ _were evaluated by using the linear regression model.

Evaluation of effects of length of infusion and infusion rate to dexmedetomidine clearance was done by using analysis of covariance. The effect of baseline patient factors on the pharmacokinetics of dexmedetomidine was tabulated by using descriptive statistics and was analyzed by patient factors. Continuous patient factors were dichotomized in descriptive tabulations by using the median as a cut-point. These patient factors included gender, age, Simplified Acute Physiology Score (SAPS II) [11] at screening, and baseline heart rate, blood pressure, creatinine, and creatinine clearance, calculated by using the Cockcroft-Gault formula [12]. The analysis-of-variance model (for categoric factors) and analysis of co-variance (for continuous factors) were used to assess statistical significance. No multiplicity adjustments were done. Statistical analyses were performed by using SAS software version 9.2 (SAS Institute Inc., Cary, NC, USA), and significance was reported at a *P *value of 0.05 or less.

## Results

### Patient population

In total, 13 Caucasian subjects were screened and included in the study (Table 1). No subjects discontinued the study prematurely, but three subjects were withdrawn from the study treatment because they died during the study. The deaths were not related to the use of dexmedetomidine. The study population consisted of both male and female subjects, with the mean age of 57.4 years (range, 18 to 82 years). The reason for ICU admission was medical in five, surgical in five, and trauma in three patients, and their mean SAPS II score was 44.8 (31.9%).

**Table 1:** Characteristics of 13 intensive care patients

	Patient number														
Variable	1	2	3	4	5	6	7	8	9	10	11	12	13	Mean	CV (%)
Weight (kg)	80	90	120	56	85	90	60	60	85	105	110	80	100	86.2	22.8
Age (years)	75	69	50	18	59	53	82	77	74	42	57	56	35	57.4	32.1
SAPS II	48	68	38	34	39	40	47	47	73	42	55	29	22	44.8	31.9
Bilirubin (μmol/L)	40	11	8	14	4	109	10	5	91	17	-	12	15	16.1^a^	138
Creatinine (μmol/L)	79	179	186	45	163	112	175	44	156	97	-	77	72	103^a^	56.2
Day from ICU admission	1.1	1.1	1.1	2.7	1.1	1.1	3.6	2.5	1.8	1.3	1.5	4.9	0.9	1.6^a^	58.3
Vasopressors	Yes	Yes	Yes	Yes	Yes	No	Yes	Yes	Yes	Yes	Yes	Yes	Yes		
Midazolam (M) or propofol (P) rate (mg/kg/h)	-	0.1 (M)	2.3 (P)	3.8 (P)	1.9 (P)	-	3.2 (P)	1.3 (P)	1.7 (P)	0.1 (P)^b^	2.9 (P)	0.1 (P)^b^	1.5 (P)^b^		

Bilirubin and creatinine values refer to the highest value during dexmedetomidine infusion. Day from ICU admission refers to the day dexmedetomidine infusion was commenced. Vasopressors indicate whether the patient received vasoactive drugs before dexmedetomidine infusion. Midazolam or propofol rate, infusion rate before the start of dexmedetomidine infusion; CV, coefficient of variation; SAPS II, Simplified Acute Physiology Score; ^a^geometric mean (backtransformed mean of the values on the logarithmic scale); ^b^intravenous boluses.

### Concomitant treatments

All patients had prior pharmacologic treatments and were already sedated before the start of dexmedetomidine infusion. Most commonly used treatments during dexmedetomidine infusion were short-acting insulin (13 of 13), oxycodone (13 of 13), noradrenaline (13 of 13), rescue propofol (12 of 13), enoxaparin (12 of 13), esomeprazole (12 of 13), and β-lactam antibacterials (11 of 13). None of the drugs used is known to be a strong inhibitor or inducer of the enzymes relevant in the pharmacokinetics of dexmedetomidine.

### Dexmedetomidine pharmacokinetics

**Figure 2:**
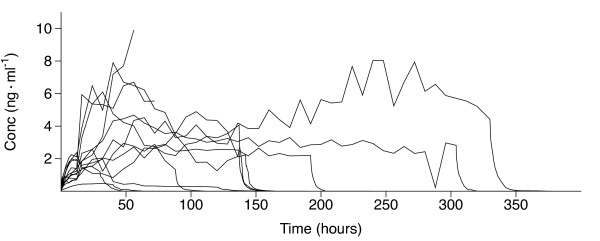
**Dexmedetomidine concentration profiles of the 13 patients during the infusion and the 48-hour follow-up**. The 48-hour follow-up was not reached in the three patients who were withdrawn from the study treatment and died of adverse events. Conc, concentration.

**Figure 3:**
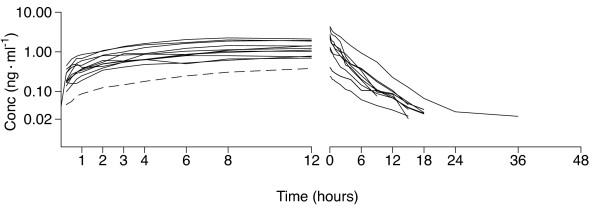
**Dexmedetomidine concentration profiles during the 12-hour constant-rate infusion (*n *= 13) and the 48-hour follow-up after stopping the infusion (*n *= 10)**. The infusion rate was 0.7 μg/kg/h in all but one patient, who received dexmedetomidine, 0.1 μg/kg/h (dashed line), during the constant-rate phase. Conc, concentration.

The observed dexmedetomidine concentrations varied widely between the patients. The individual dexmedetomidine plasma concentration-versus-time curves during the entire study period are shown in Figure 2. The corresponding curves during the initial commencement and after the discontinuation of the infusion are depicted in Figure 3.

The values of the pharmacokinetic variables are shown in Table 2. The geometric mean value of dexmedetomidine clearance was 39.7 L/h (40.9%). In the 10 patients with complete data sets, the geometric mean values for *t*_1/2_, Cl, and V during the elimination phase were 3.7 h (38.1%), 41.4 L/h (46.7%) and 223.3 L (35.3%), respectively.

**Table 2:** Characteristics and pharmacokinetic parameters of dexmedetomidine infusions in 13 intensive care patients

	Patient number														
Variable	1	2	3	4	5	6	7	8	9	10	11	12	13	Mean	CV (%)
Rate of the 12-h initial infusion (μg/kg/h)	0.1	0.7	0.7	0.7	0.7	0.7	0.7	0.7	0.7	0.7	0.7	0.7	0.7	0.6^a^	58.1
Maximum infusion rate (μg/kg/h)	0.1	2.1	2.5	2.5	2.5	2.5	2.5	0.95	0.7	2.5	1.2	2.5	2.5	1.5^a^	117
Length of infusion (h)	40	142	137	88	55	304	137	30	121	330	13	72	192	92.0^a^	117
Cumulative dose (mg)	0.34	11	25	8.7	8.0	56	17	1.4	2.4	82	1.1	13	46	21.0	120
AUC_0-∞ _(ng h/ml)	17.8	376	550	174	252	924	337	39.0	100	1,530	29.7	350	583	213^a^	233
Extrapolated fraction of AUC_0-∞ _(%)	1.3	0.1	0.0	0.1	22.0	0.0	0.0	0.6	0.2	0.0	37.1	9.3	0.0		
*t*_1/2 _(h)	4.5	4.2	2.9	5.0	-	2.4	3.4	3.3	6.9	5.1	-	-	2.1	3.7^a^	38.1
Cl (L/h)	18.9	30.4	45.4	50.4	31.8	60.7	51.5	35.5	23.7	53.7	36.1	36.7	79.0	39.7^a^	40.9
V (L)	123	184	193	362	-	213	252	170	234	391	-	-	234	223^a^	35.3

Because of missing samples for subjects 5, 11, and 12, it was not possible to define the elimination-phase parameters for these subjects, and the area under the dexmedetomidine concentration-time curve extrapolated to infinity (AUC_0-∞_) was estimated by using the mean elimination half-life of the other patients. Subject 5 received hemodiafiltration. Cl, plasma clearance; CV, coefficient of variation; *t*_1/2_, elimination half-life; V, volume of distribution during elimination; ^a^geometric mean (backtransformed mean of the values on the logarithmic scale).

**Figure 4:**
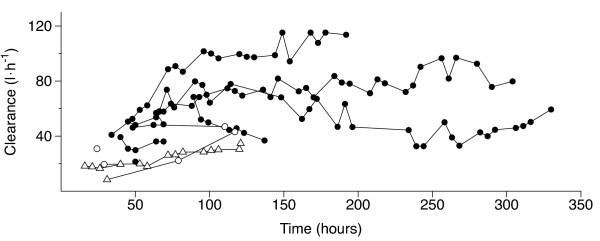
**Dexmedetomidine clearance at 116 steady states defined by a 15-hour constant-rate infusion in 12 subjects**. Open triangles, open circles, and solid circles indicate an infusion rate of 0.1, 0.4 to 2.1, and 2.5 μg/kg/h, respectively. Each line represents one patient. In two patients, only one steady state was achieved, and the corresponding clearances are depicted with a single symbol.

There were 116 steady-state concentrations in 12 subjects (Figure 4). Fifteen of the steady-state concentrations were reached during an infusion rate of 0.1 μg/kg/h, one during an infusion rate of 0.4 μg/kg/h, three during an infusion rate of 0.7 μg/kg/h, one during an infusion rate of 2.1 μg/kg/h, and 96 during an infusion rate of 2.5 μg/kg/h. The geometric mean value for Cl_ss _was 53.1 L/h (54.8%; range, 8.4 to 115 L/h). A statistically significant relation was found between dexmedetomidine Cl_ss _and length of infusion (*P *= 0.0007) and infusion rate (*P *< 0.0001) in univariate analysis. In multivariate analysis, where both length of infusion and infusion rate were included in the model, only the infusion rate was statistically significant (*P *< 0.001). The interaction between the length of the infusion and the infusion rate was not statistically significant.

**Figure 5:**
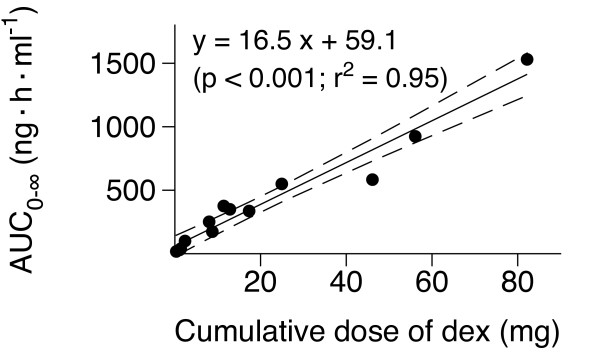
**Relation of the total dexmedetomidine dose and the area under the dexmedetomidine concentration-time curve extrapolated to infinity (AUC)**. The dashed lines represent the 95% confidence intervals for the regression line (solid line).

The AUC_0-∞ _of dexmedetomidine plotted against the total cumulative dose is shown in Figure 5. A statistically significant linear relation (*r*^2 ^= 0.95; *P *< 0.001) was found between AUC_0-∞ _and the cumulative dose of dexmedetomidine.

The dexmedetomidine Cl was higher (*P *= 0.006) in patients with low baseline SAPS II scores (< 42) compared with subjects with high scores (≥ 42). The corresponding values for geometric mean Cl were 57.6 L/h (24.7%) and 33.2 L/h (43.5%), respectively. In subjects with low baseline SAPSII scores (< 42), dexmedetomidine *t*_1/2 _was shorter (*P *= 0.036) compared with subjects with higher baseline SAPSII scores (≥ 42), with geometric mean values being 2.9 hours (39.9%) and 4.4 hours (27.6%), respectively. Dexmedetomidine Cl and creatinine clearance showed a linear correlation at baseline (*P *= 0.026). Other patient factors did not significantly affect clearance.

### H-3 metabolite pharmacokinetics

The geometric mean value for *t*_1/2 _of H-3 during the elimination phase was 9.1 hours (37.0%; *n *= 10). The geometric mean ratio of AUC_0-∞ _of H-3 metabolite to that of dexmedetomidine was 1.47 (105%), ranging from 0.29 to 4.4. The ratio was not statistically significantly related to the total dose of dexmedetomidine (*P *= 0.528) or the duration of the infusion (*P *= 0.872).

### Effect of hemodiafiltration

Hemodiafiltration was performed for one subject who was admitted with acute on chronic renal failure and had renal-replacement therapy during the current admission. Based on simultaneously measured drug concentrations in the afferent and efferent limbs of the filter circuit, no evidence was found in this single subject that dexmedetomidine or H-3 metabolite was extracted from plasma by hemodiafiltration (data not shown).

### Depth of sedation

**Figure 6:**
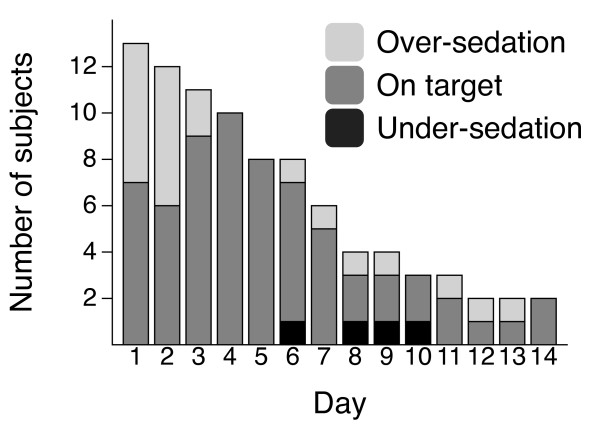
**Success in reaching the target Richmond Agitation-Sedation Scale**.

Altogether 88 RASS values were recorded in the 13 patients. In four of the 88 recordings (4.5%), undersedation was discovered, when the patients were treated according to the protocol (dexmedetomidine infusion enhanced with propofol infusion or boluses). All but one patient (12 of 13) needed at least one bolus dose of propofol to keep the RASS value in the target zone, and the median amount of propofol was 1,869 mg/day (range, 79 to 8,505 mg). The number of patients oversedated, on target, and undersedated during the study is shown in Figure 6.

### Safety

In total, 53 adverse events were reported in 12 subjects after the start of the study treatment. The most common adverse events were tachycardia (nine events), hypotension (five events), and hypertension (four events). Four of the adverse events were assessed as being related to the study treatment: three episodes of bradycardia and one episode of first-degree AV block. These four adverse events all resolved, with the bradycardias requiring dexmedetomidine dose reduction, and, in two cases, administration of atropine and glycopyrrolate. Most of the adverse events were recorded in the highest dose range of 1.4 to 2.5 μg/kg/h, but no clear correlation was noted between the onset of the adverse events and the dexmedetomidine concentration in plasma.

Twenty of the 53 adverse events, reported in seven of the 13 subjects, were classified as serious adverse events. Five of the serious adverse events led to death, with three of the deaths occurring during the study-drug infusion period and leading to discontinuation of the study treatment. The causes of the deaths were chronic pulmonary disease (*n *= 2), sepsis, myocardial infarction with a myocardial rupture, and pancreatitis.

## Discussion

A strong linear relation was found between the area under the dexmedetomidine concentration-time curve and the cumulative dexmedetomidine dose in critically ill patients. This suggests that the pharmacokinetics of dexmedetomidine is linear at least up to the dose of 2.5 μg/kg/h, even in this very sick patient population, with mean SAPS II of 44.8 (range, 22 to 73). This characteristic means that when the dose of dexmedetomidine is increased, its concentrations increase in a linear manner with consequent potential pharmacologic effects [13].

Previous studies assessing the pharmacokinetics of dexmedetomidine have involved mostly healthy subjects [2,14,15]. One previous report exists on the pharmacokinetics of dexmedetomidine during ICU treatment in postoperative coronary artery bypass patients, but the dose in that study was limited to a maximum of 0.7 μg/kg/h, and the mean duration of treatment was 10 hours (range, 6 to 16 hours) [6]. The elimination half-life of dexmedetomidine after the long duration of treatment in our patients was comparable but still somewhat longer than reported previously for dexmedetomidine infusion of shorter duration and in healthier patients or volunteers [2,6,15]. However, the values for dexmedetomidine clearance and volume of distribution were similar. The slower elimination of dexmedetomidine, as judged by means of the elimination half-life, may be due to the critical condition of our patients.

In one patient, the concentration of dexmedetomidine (Figure 2) increased exceptionally during the 24-hour period before her death from 3.4 to 9.9 ng/ml, with no signs of liver dysfunction. She had paralytic ileus and severe septic shock, and her cardiac index was very low (0.4 to 1.3 L/min/m^2^) during her last day of life. Her postmortem examination revealed myocardial infarction with a rupture of the myocardium and hemopericardium to be the direct causes of death. According to previous studies [3,16], the clearance of dexmedetomidine may depend on hepatic blood flow. In this case, it can be reasoned that dexmedetomidine concentration increased exceptionally because of the impaired liver blood flow.

A Cl_ss _could be calculated on 116 occasions during dexmedetomidine infusions. Although the rate of infusion was statistically significantly associated to Cl_ss _in multivariate analysis, it is unlikely that dexmedetomidine would exhibit nonlinear pharmacokinetics. The increase in dexmedetomidine Cl_ss _with the rate of infusion may be related to improvement of the general condition of the patients; that is, the sickest patients with poor liver flow are more likely to need low doses. When a patient is getting better with time with a simultaneous increase of the liver blood flow and improvement of dexmedetomidine clearance, he/she needs more sedative agents, thus giving the false impression of an association between dexmedetomidine clearance and the dose.

One of the secondary objectives of the study was to evaluate the patient factors that affect the pharmacokinetics of dexmedetomidine. The mean dexmedetomidine clearance was statistically significantly higher, and the mean elimination *t*_1/2 _statistically significantly shorter in subjects with a baseline SAPS II score < 42, as compared with the subjects with baseline SAPS II score > 42. In addition, a linear correlation appeared between dexmedetomidine clearance and baseline creatinine clearance. This may suggest a relation between the general condition of the patient and lower dexmedetomidine clearance, because renal function itself does not affect pharmacokinetics of dexmedetomidine [17]. None of the other patient factors that we evaluated significantly affected the pharmacokinetic parameters of dexmedetomidine. Hemodiafiltration was performed on one subject during the study. No evidence was noted in this single subject that dexmedetomidine was extracted from plasma by hemodiafiltration. This finding must be confirmed in further studies.

The geometric mean value of the *t*_1/2 _of H-3 metabolite was 9.05 hours, which is longer than that of the parent dexmedetomidine, and the geometric mean ratio of AUC_0-∞ _of H-3 metabolite to that of dexmedetomidine was 1.47 (105%). However, the affinity of the H-3 metabolite for α-2 receptors is less than 0.5% that of dexmedetomidine (data on file) and therefore is not expected to have clinically significant pharmacologic effects at clinically relevant dexmedetomidine doses.

In this study, the patients were administered dexmedetomidine to achieve a predefined level of sedation. A significantly higher dose than described in the summary information provided by the manufacturer was allowed, and doses up to a maximum rate of 2.5 μg/kg/h were administered to most of the patients. Indeed, approximately 70% of the entire study duration was spent at the highest dose range (1.4 to 2.5 μg/kg/h). This dosing regimen provided mostly adequate sedation, although practically all patients received also additional propofol (dose range, 79 to 8,505 mg/day). However, for two of the three patients receiving the highest doses of additional propofol, the RASS target was amended to -4 for most of the study duration, indicating that they required deeper sedation. The high propofol use in these patients is in accordance with earlier studies, which indicated that dexmedetomidine alone may not be sufficient in patients requiring deep levels of sedation [18].

Surprisingly, tachycardia was the most common adverse event reported after start of the study treatment, even though dexmedetomidine is well known to cause bradycardia. However, tachycardia is common in ICU patients [19], and the incidence of tachycardia and bradycardia depend on the definition of them. Nevertheless, we do not believe that dexmedetomidine *per se *induces tachycardia.

Four adverse events were assessed to be related to the study treatment. These included three incidents of bradycardia and one episode of first-degree AV block. Dexmedetomidine has been shown to have significant effects on cardiac conduction in pediatric patients, possibly related to a decrease of sympathetic tone in the CNS and/or a reflex response to the systemic vasoconstriction caused by dexmedetomidine [20].

There have been inconsistent results in previous studies on the effect of dexmedetomidine dose on the incidence of adverse events. Some studies suggest that higher doses of dexmedetomidine may result in a higher incidence of hypotension or bradycardia, whereas some studies suggest the opposite [21]. In our study, most of the adverse events during the study treatment were recorded at the highest dose level (> 1.4 to 2.5 μg/kg/h), but no clear correlation was seen between the onset of the adverse events and the dexmedetomidine concentration in plasma. This finding was to be expected, because most of the study duration was spent at this highest dose range, and most adverse events were likely related to the underlying critical illness. However, the effect of dexmedetomidine dose on the incidence of adverse events must be confirmed in future studies. Serious adverse events seen in this study were typical of critically ill patients, and none of them was considered related to dexmedetomidine.

This study has several limitations. The small number of patients limits further generalization of the results. Patients having severe hepatic impairment (bilirubin > 101 μmol/L) were not considered eligible for the study, and therefore, the results cannot be extrapolated to patients with severe hepatic dysfunction. Although we excluded also patients with uncompensated circulatory failure at screening, many of the patients developed various circulatory problems during the study. Some of the observed variability in the pharmacokinetics of dexmedetomidine may thus be related to reduction in liver blood flow.

## Conclusions

This study describes the pharmacokinetics of high-dose dexmedetomidine in a critically ill long-stay ICU patient population. The results suggest that dexmedetomidine obeys linear pharmacokinetics up to the dose of 2.5 μg/kg/h, and dexmedetomidine clearance is lower in patients with high baseline SAPS II scores than in those with low SAPS II scores. Despite the high dosing regimen and prolonged infusions, no new safety findings were found for dexmedetomidine.

## Key messages

• Dexmedetomidine appears to have linear pharmacokinetics up to the dose of 2.5 μg/kg/h.

• Dexmedetomidine clearance is lower in patients with high baseline SAPS II scores than in those with low SAPS II scores.

• Despite the high dosing regimen and prolonged infusions, no new safety findings were noted for dexmedetomidine.

## Abbreviations

AUC: area under the drug concentration-time curve; Cl: plasma clearance; Cl_ss_: plasma clearance at steady state; C_ss_: concentration at steady state; HR: heart rate; ICU: intensive care unit; I_ss_: infusion rate at steady state; *k*_e_: elimination rate constant; MAP: mean arterial blood pressure; RASS: Richmond agitation-sedation scale; SAPS II: simplified acute physiology score; *t*_1/2_: elimination half-life; V: volume of distribution during elimination.

## Competing interests

Dr Iirola has received speaker fees from Orion Corporation (Espoo, Finland). Dr Aantaa has been a paid consultant for Orion Corporation (Espoo, Finland) and Abbott Laboratories (Abbott Park, IL, USA), the original co-developers of dexmedetomidine, as well as for Hospira (Lake Forest, IL, USA). Hospira has a license agreement with Orion Corporation concerning dexmedetomidine (Precedex). Dr Lahtinen has been engaged in contract research for Orion Corporation (Espoo, Finland). Drs Laitio, Kentala, and Professor Olkkola have no conflicts of interest that are directly relevant to the content of the study. Mr Wighton, Dr Garratt, and Ms Ahtola-Sätilä are all employees of Orion Pharma.

## Authors' contributions

TI, CG, TA-S, KTO, and AW designed and performed research, analyzed data, and drafted the manuscript. RA designed research, analyzed data, and drafted the manuscript. RL and EK designed and performed research and drafted the manuscript. ML analyzed dexmedetomidine and H-3 samples. All authors read and approved the final manuscript for publication.
